# Dr. Goodhue’s Address before the Rock River Medical Society

**Published:** 1846-08

**Authors:** 


### ARTICLE XV.

### DR. GOODHUE'S ADDRESS BEFORE THE ROCK RIVER MEDICAL SOCIETY.

The address of J. C. Goodhue, M.D., delivered at the first annual meeting of the Rock River Medical Society, although of a popular character, to adapt it to the audience present on the occasion, offers points of interest to every physician in this region. The Doctor was one of the earliest regular medical practitioners in this part of the country, and his remarks upon the date of the profession here, are worthy of recollection.

He says: "When I first commenced the practice of Physic and Surgery, in the then village of Chicago, in eighteen hundred and thirty three, for it was at that time but a small insulated village, about one third the present size of Rockford, there was not more than two white settlers, and not one physician within the limits over which this society proposes to extend its influence; yes, gentlemen, from Dixon to Janesville, from Freeport to Pleasant Grove, John Dixon and Stephen Mack were, as far as I am informed, the only white men who had pushed their way into this beautiful and now fertile and well settled portion of the Rock River Valley.—p. 2.

"A few moments are due to the early medical pioneers. So far as I am informed, Doctor Harmon was the first physician who settled at Chicago. There had undoubtedly been surgeons of the United States Army stationed at Fort Dearborn during the war of eighteen hundred and twelve, but Dr. Harmon was the pioneer among the medical faculty of this corner of Illinois. I am informed he still lives, and is at Naperville, in Du Page county. Dr. Kimberly was the second, and is still among the most respectable practitioners of Chicago. Doctor John T. Temple, of St. Louis, and Doctor Clark, since dead, were next. Doctors Egan, Eldridge, and myself soon followed at about the same time. This brings it to the spring of thirty-four, when a perfect flood of emigration poured in, and among them a sprinkling of medical men. Many of the early settlers of Chicago are still in the field of medicine, and engaging the confidence of the public."—p. 3.

"Within the bounds which this society propose to include, within a circuit bounded on the north by Janesville, south by Dixon, east by Pleasant Grove, and west by Freeport, Doct.

D. H. Whitney, as far as I am informed, was the first physician. He settled at Squaw Prairie, now Belvidere, as early as thirty-five or six, and has continued to reside there to the present time. Doctor George Dunbar was for a short time at Rockford, in thirty-six, and was, I believe, the second practitioner within the before named limits. Doctors Hall, Haskell and Goodrich were here at an early day, also Doctor Malony at Belvidere, Doctor White at Beloit, Doctor Van Valzeh at Freeport, and many others."—p. 4.

In reference to the characters and motives of those early physicians we record the following, as it may be applicable to the cases of some who are coming here at present.

"Anterior to eighteen hundred and forty, nine-tenths of all the physicians who had located themselves in this region, had done so with reference to pursuing agriculture, and with the avowed intention of abandoning medical practice; most of whom, either from the necessity of the case, or from finding more truth than poetry in pounding out rails, resumed their profession, and divided their attention between farming and medicine."—p. 4.

He does not dwell at length upon the character of the diseases or their treatment, but only alludes occasionally to these points.

"The diseases that have heretofore prevailed in the country, have been but a few from the long catalogue, and those few, easy of treatment, and requiring little variation in practice. Intermittent and remittent fevers, Diarrhœa, Dysentery, Pneumonia, will include nearly all the ills flesh has been heir to here as yet, and these in their mildest forms. This fact, alone, can account for the general good success of these farming doctors."—p. 5.

The scope of this address embraces the whole field of the duties of the physician, his studies and his conduct, and exhibits with vigor, clearness, and at times with eloquence, the scenes he must pass through, and the character he should sustain as a member of a time honored and liberal profession. It portrays in its true colors, all modern forms of quackery. The sentiments of it were heartily responded to by the members of the society, in such a manner as to show that they were heartily approved, and to indicate that among all the changes to which we are subjected, there still remains among the working men of the medical profession, a little of the good old spirit, felt and transmitted by Gregory, Percival, Rush, and a crowd of worthies whose names adorn the pages of medical history.

